# Meckel's diverticulum: analysis of 27 cases in an adult population

**DOI:** 10.3389/fsurg.2023.1327545

**Published:** 2023-12-18

**Authors:** Juan David Hernández, Gustavo Valencia, Felipe Girón, Andrés Mauricio García Sierra, Ricardo E. Núñez-Rocha, Lina M. Rodríguez, Carlos Eduardo Rey Chaves, Eduardo Emilio Londoño, Ricardo Nassar

**Affiliations:** ^1^ Department of Surgery, Hospital Universitario Fundación Santa Fe de Bogotá, Bogotá, Colombia; ^2^ School of Medicine, Universidad de los Andes, Bogotá, Colombia; ^3^ School of Global Health Management and Informatics, University of Central Florida, Orlando, FL, United States; ^4^ Estudiante de posgrado Cirugía General, Pontificia Universidad Javeriana, Facultad de Medicina, Bogotá, Colombia

**Keywords:** Meckel's diverticulum, surgery, laparoscopy, diagnosis, Latin America

## Abstract

**Background:**

Meckel's diverticulum is a rare congenital pathology among newborns. Nevertheless, it is an uncommon abdominal pathology in the adult population. Therefore, we aim to provide a detailed account of our surgical approach in treating 27 cases of Meckel's diverticulum.

**Methods:**

This study is a cross-sectional analysis that utilized a database with prospectively collected data from 2004 to 2022. All patients under the age of 18 were excluded from the population. We described the population’s demographic characteristics, symptoms, anatomopathological study, surgical technique, complications, morbidity, and mortality. A subgroup analysis was performed between the symptomatic and asymptomatic patients.

**Results:**

A total of 27 patients who underwent surgical resection for a posteriorly diagnosed Meckel's diverticulum were included. The male population accounted for 81.4% (*n* = 22) of the sample size. The symptomatic group consisted of 18 male and four female patients. Abdominal pain was the predominant symptom in 85% of the patients. Out of the 22 symptomatic patients, only 9% had a positive perioperative diagnosis of Meckel's diverticulum. All 27 patients with diverticulum diagnosis received the resection through diverticulectomy (*n* = 6), small bowel resection with end-to-end anastomosis (*n* = 6), and small bowel resection with lateral to lateral anastomosis (*n* = 15). The mean distance between the diverticulum and the ileocecal valve was 63.4 cm. The symptomatic group had an average diverticulum length of 3.54 cm, with an average base width of 2.47 cm. In the other group, the values were 2.75 and 1.61 cm. The average length of hospital stay in the symptomatic group was 7.3 days.

**Conclusions:**

Meckel's diverticulum is a rare pathology in the adult population. Its presentation varies from asymptomatic to symptomatic patients, and surgery is the cornerstone treatment for this pathology.

## Introduction

Approximately in the fourth to sixth week of gestation, the yolk sac and the primitive midgut are connected by the “Vitello” intestinal duct. By the fifth to seventh week, this intestinal duct is normally obliterated; when this process fails, it can lead to numerous abnormalities such as omphalomesenteric fistula, omphalomesenteric cyst, and the most common one, Meckel's diverticulum, which occurs in approximately 2%–4% of the cases (1, 2). It is an uncommon abdominal pathology in adults, often described with the “rule of twos”: occurring in 2% of the population, located approximately 2 ft from the ileocecal valve, measuring about 2 inches in length, being frequently observed in children under 2 years of age, and affecting males twice as often as females (3, 4). It has been classically estimated that one in every 300–400 cases of acute abdomen in adults is caused by a complicated Meckel's diverticulum (5). However, the lifetime risk of complications has been estimated to decrease from 3.7% at 16 years to 0.42% at 86 years (6) or, as described more often, a 4% risk throughout one’s lifespan to almost zero at age 80 (7). Specifically, in adults, the most frequent complications include bowel obstruction due to intussusception or adhesive band (14%–53%), ulceration, diverticulitis, and perforation (4). A diagnosis of Meckel's diverticulum requires a high index of suspicion, and even with the use of modern imaging technologies, they are often diagnosed intraoperatively (8). Controversy surrounds the surgical treatment, which will depend on the etiology of the complication (9) and the clinical characteristics of the patient. Furthermore, the appropriate course of action when an asymptomatic diverticulum is incidentally discovered during surgery for other causes is also a matter of discussion (9). To date, literature is lacking regarding the clinical characteristics and outcomes of patients with Meckel's diverticulum in the adult population in Latin America; therefore, this article aims to describe the clinical features and surgical outcomes of 27 symptomatic and asymptomatic incidentally found cases seen in a fourth-level hospital in Colombia.

## Materials and methods

### Study population

A cross-sectional study utilizing prospectively collected data from computerized databases was conducted with the approval of the Institutional Review Board and following the guidelines established by the Health Insurance Portability and Accountability Act (HIPAA). The study focused on the reports of histopathologic examinations performed at a fourth-level hospital. In the last 12 years, these reports were reviewed searching for those containing Meckel's diverticulum in their diagnosis. All reports of patients under 18 years were excluded. In addition, patients who did not have available or accessible clinical records were excluded from the study. The study adhered to the ethical guidelines outlined in the Helsinki Declaration and complied with current local legislation on research.

### Data management

The clinical charts were reviewed to document data on age, sex, symptoms, histopathology, surgical technique, morbidity, and mortality. Diagnostic imaging exams used on each patient were also recorded. The study also recorded the surgical approach and its relationship with early and late complications, as well as the length of hospital stay. A laparoscopic approach was defined as the event in which the entire procedure was completed within the abdominal cavity, and a laparoscopic-assisted approach corresponded to the surgery in which the intestine was exteriorized off the abdominal cavity using a small incision after a laparoscopic approach to complete the resection. The distance of Meckel's diverticulum from the ileocecal valve was used when available. For further analysis, the patients were categorized into two groups: asymptomatic and symptomatic. The asymptomatic group consisted of patients who underwent surgery for a different indication and a Meckel's diverticulum was discovered incidentally. The histopathology was also recorded for these two groups. Pathologists conducted a new observation of all the available histological slides. The presence of ectopic tissue and the length of the diverticulum were documented. The morbidity was categorized into early and late complications after the initial surgery. An early complication was defined as a condition secondary to the operation appearing during the first 30 days, while a late complication was considered a condition that occurs after 30 days.

### Statistical analysis

Descriptive statistics were provided for all variables based on their nature. The continuous variables were summarized by means of medians and standard deviation or interquartile ranges, according to their nature and distribution (determined by the Kurtosis/Skewness test). Dichotomous variables were described using frequency and proportion.

## Results

### Patients

**Figure 1:**
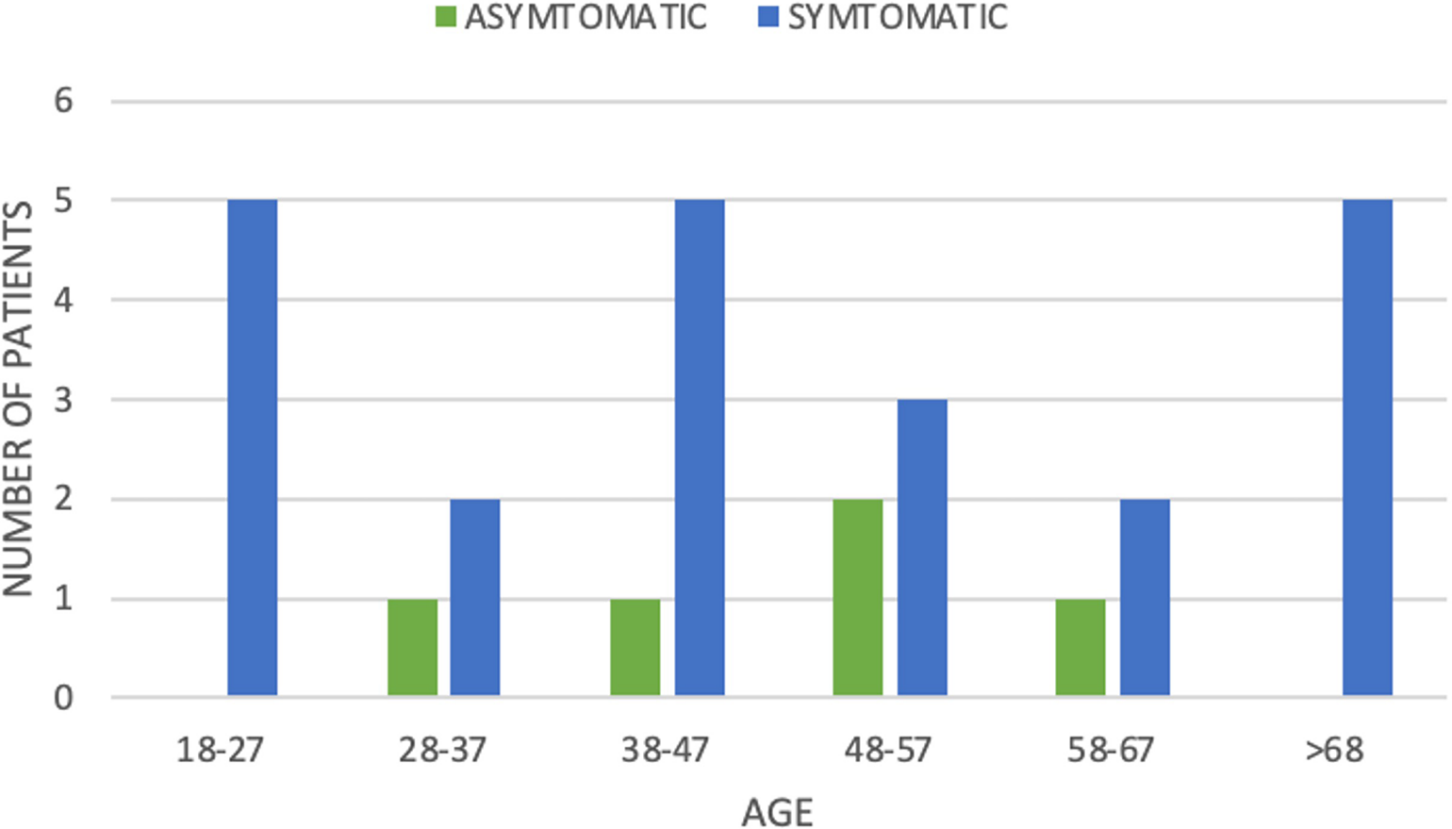
Number of patients according to age and symptomatic vs. asymptomatic Meckel's diverticulum.

From January 2004 to June 2016, a total of 42 pathology reports included the diagnosis of Meckel's diverticulum. Out of these, 15 reports with patients under the age of 18 were excluded. A total of 27 reports involving adult patients were retrieved (see Table 1). There were 22 males and five females in this set of patients, resulting in a male-to-female ratio of 4.4:1. In the asymptomatic group, four males and one female, between 20 and 66 years old, had an incidentally found Meckel's diverticulum resected during an operation. None of these patients had exhibited any prior symptoms that could be attributed to Meckel's diverticulum. The symptomatic group consisted of 18 males and four females, between 18 and 82 years, with a male-to-female ratio of 4.5:1. The age distribution of the patients is seen in Figure 1.

**Table 1:** Demographic characteristics of the subject population.

Demographic characteristic	Symptomatic (*n*)	Asymptomatic (*n*)
Patients	22	5
Sex		
Men	18	4
Women	4	1
Age range (years)	18–82	20–66
Mechanism of disease		
Bleeding	3	—
Obstruction	13	—
Inflammation	5	—
Perforation	1	—
Histopathology		
Gastric ectopic mucosa	9	—
Normal ileal mucosa	13	5
Inflammation of mucosa	22	5
Surgery		
Totally laparoscopic	8	2
Lap-assisted	8	—
Laparotomy	6	3
Early morbidity		
Medical treatment	15	3
Re-operation	2	—
Late morbidity		
Medical treatment	1	—
Re-operation	2	—

### Symptoms

**Figure 2:**
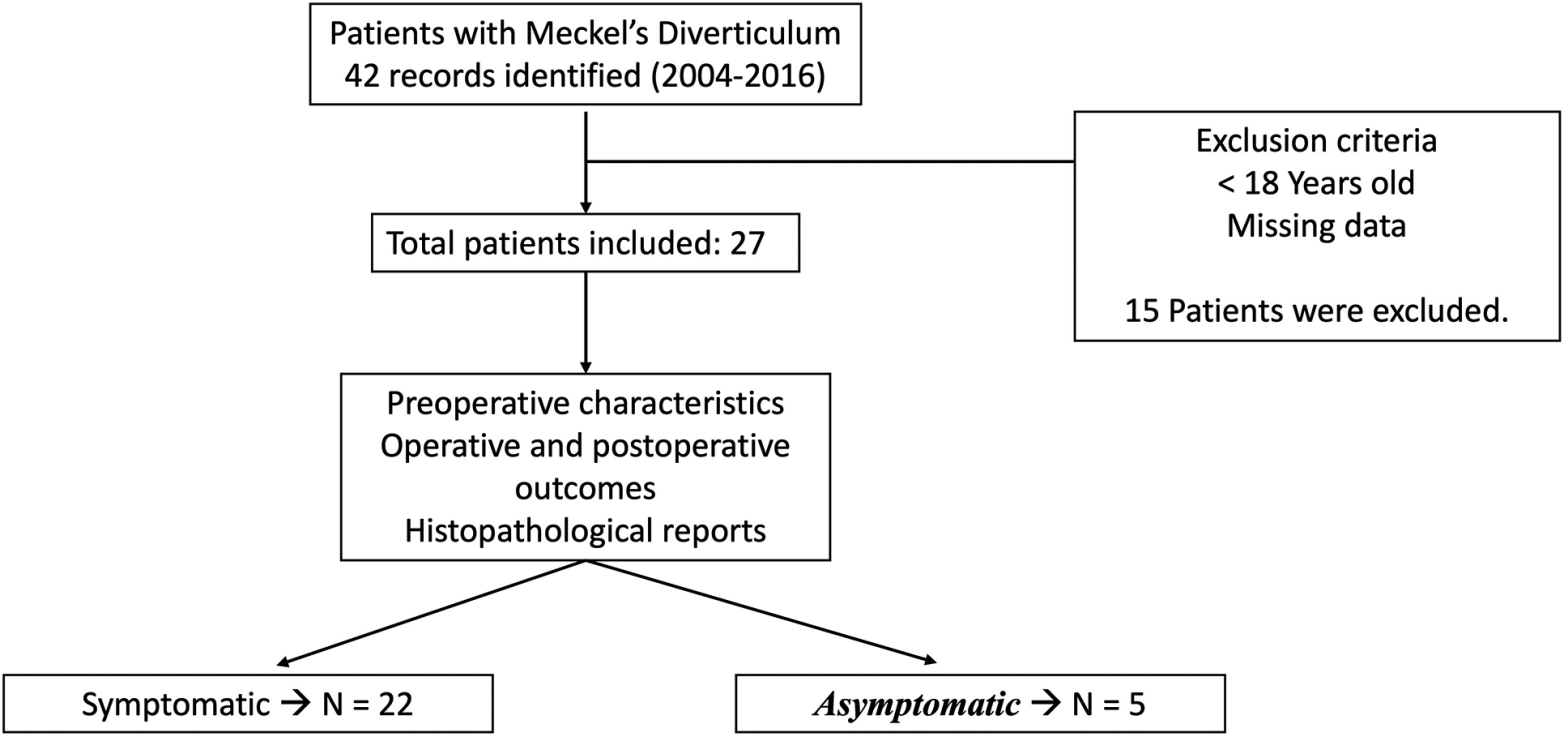
Flowchart of the method.

The 22 patients in the symptomatic group presented with a variety of symptoms (see Table 2). Among them, 82% appeared with two or more symptoms at the emergency room, and only three patients experienced abdominal pain only. Abdominal pain was the predominant symptom, leading to 85% of them seeking medical attention in the emergency room. It is also the most common symptom in patients with small bowel obstruction (SBO) and perforation, with a 100% rate of presentation. All five asymptomatic patients were taken to surgery due to a condition different from Meckel's diverticulum: two patients presented with a variety of symptoms due to appendicitis, two patients were scheduled for a ureter-ileostomy after a cystectomy and the other one had a GIST in the jejunum. Hence, Meckel's diverticulum was not producing symptoms, and the diverticulum was found incidentally (Figure 2).

**Table 2:** Symptoms in complicated Meckel's diverticula patients.

Symptom	Number of patients
Abdominal pain	22
Vomit	10
Diarrhea	6
Nausea	4
Asthenia	3
General discomfort	3
Hematochezia	2
Dizziness	2
Hyporexia	2
Melena	2
Fever	2
Rectal bleeding	1
Syncope	1
Total symptomatic patients	22

### Diagnosis

As is done in the evaluation of a patient with acute abdominal pain, a variety of tests were conducted to establish the cause of the patient's symptoms (see Table 3). Out of the 22 symptomatic patients, only two (9%) received a positive diagnosis made of Meckel's diverticulum by preoperative scans (^99^mTc scintigraphy), which were ordered to establish the cause of hemorrhage. The studies confirmed the presence of ectopic gastric mucosa. In another patient, there was a high index of suspicion prior to the operation based on the CT scan findings of ileitis with a high likelihood of Meckel's diverticulitis. This finding was later confirmed by pathologic examination. Among the remaining 12 patients in the symptomatic group (54%), an abdominal CT scan confirmed the presence of SBO, but the probable cause was not reported.

**Table 3:** Diagnostic exams.

Diagnostic exam	Number of patients
Abdominal CT	16
Abdominal x-ray	12^a^
Abdominal ultrasound	6
Colonoscopy	3
Capsule endoscopy	2
Endoscopy	2
Ectopic gastric mucosa scan	2
Labeled red blood cell scan	1
Urography CT	1

^a^ Two abdominal x-rays were performed on the same patient, and in another patient, an abdominal x-ray was performed at the referring hospital and another one upon arrival.

On the other hand, six patients from the complete study population (27.7%) were diagnosed preoperatively with appendicitis and taken to surgery. Two of them were assigned to the asymptomatic group due to the incidental discovery of the diverticulum, while the other four patients were included in the symptomatic group since the symptoms were attributed to Meckel's diverticulum rather than the appendix. Among the latter, two patients experienced diverticulum perforation, while the other patient had diverticulitis. In one of them, the appendix was found to be in a normal state and was left *in situ*, while the diverticulum was resected. Another symptomatic patient exhibited inflammation of the appendix in addition to a perforated Meckel's diverticulum, necessitating the resection of both.

### Surgery

All 27 patients had the diverticulum resected using different surgical techniques (see Table 4), according to surgeons’ preferences and due to the clinical presentation. The surgeon selected a surgical approach (open or laparoscopic) based primarily on predilection, although in some cases taking into account the patient's clinical condition. A detailed summary of the different approaches used is provided in Table 5.

**Table 4:** Surgical techniques used to treat symptomatic Meckel's diverticulum.

	Diverticulectomy	Small bowel resection with end-to-end anastomosis	Small bowel resection with lateral–lateral anastomosis
Symptomatic			
Hemorrhage	1	1	1
Obstruction	1	2	10^a^
Perforation	1	—	1^a^
Inflammation	—	—	3
Asymptomatic	3^b^	3	—
Total	6	6	15

^a^ One patient in each group had manual suture reinforcement after stapling.

^b^ One patient had a manual diverticulectomy with a two-layer closure of the defect, and the other two patients had resection of the diverticulum with mechanical stapler.

**Table 5:** Laparoscopy vs. laparotomy in the treatment of Meckel's diverticulum.

	Laparoscopy	Lap-assisted	Laparotomy
Symptomatic			
Hemorrhage	1	1	1
Obstruction	5	2	7^a^
Perforation	—	2	—
Inflammation	—	3	—
Asymptomatic	2	–	3
Total	8	8	11

The indication for surgery is also described.

^a^ Two patients were converted from laparoscopy to laparotomy due to the presence of dilated bowel as it made visualization difficult.

### Distance from the ileocecal valve

The distance of Meckel's diverticulum from the ileocecal valve was measured in 19 out of the 27 patients. Four cases were incidentally found, and 15 were symptomatic patients. The mean distance measured was 63.4 cm with a range between 20 and 100 cm.

### Diverticular dimensions

The dimensions of diverticula varied based on whether they were symptomatic or incidentally found. The symptomatic group had an average length of 3.54 cm, ranging from 0.5 to 7.0 cm. The average base width was 2.47 cm (ranging from 0.3 to 5.0 cm). In the other group, the average length was 2.75 cm (range: 1.5–5.0 cm), and the average base width was 1.61 cm (range: 1.0–2.0 cm).

### Pathology

The pathologic specimens of 17 patients were reviewed again by pathologists. Five of the specimens could not be retrieved, and their original report was included in this study. Eight of the symptomatic diverticula (47%) presented gastric mucosa within the diverticulum. Ectopic mucosa was observed in all three patients with hemorrhage, four out of nine patients with obstruction, and one out of two patients with perforation. On the other hand, none of the five asymptomatic patients had ectopic tissue. Additional pathological characteristics found in the diverticula are presented in Table 6.

**Table 6:** Abnormal characteristics found in the microscopic examination of Meckel's diverticula.

	Microscopic characteristics		
	Inflammation	Mucosal hemorrhage	Ulcer
Symptomatic			
Hemorrhage	3	1	2
Obstruction	8	4	1
Perforation	2	1	2
Diverticulitis	3	2	1
Asymptomatic	2	—	—
Total	18	8	6

### Length of stay

The average length of hospital stay in the symptomatic group was 7.3 days, with a range spanning from 2 to 24 days. The group of patients who presented with SBO as a complication of Meckel's diverticulum had a longer average length of stay of 9.9 days, with a range spanning from 4 to 24 days. In comparison, the patients with hemorrhage and inflammation had much shorter length of stay, with an average duration of 3 days (ranging from 2 to 4 days) and 3.6 days (ranging from 3 to 4 days), respectively. In contrast, in the asymptomatic group of patients, the average length of stay was 5.6 days, with a range spanning from 1 to 10 days. Patients undergoing laparoscopic procedures had a shorter duration of hospitalization compared with those who have open procedures (4.5 days, range: 3–10 days, vs. 7.3 days, range: 4–24 days).

### Morbidity

Early complications are listed in Table 7. Out of the patients that required blood transfusions (4, 2, and 1 blood bags), two patients were diagnosed with hemorrhage caused by Meckel's diverticulum. One of these patients required vigorous fluid reanimation due to his hypovolemic shock at admission to the emergency room, and the other patient required a blood transfusion preoperatively but was not in hypovolemic shock. Another case corresponds to the incidentally found group and required blood transfusion intraoperatively.

**Table 7:** Early complications in patients with Meckel's diverticulum.

Complication	Number of patients
Hypokalemia	9
Diarrhea	7
Paralytic ileus	5
Anemia requiring blood transfusion	5
Hypomagnesemia	5
Hypocalcemia	4
Vomit	4
Hyponatremia	3
Difficult control pain	3
Nosocomial pneumonia	2
Generalized peritonitis	2
Bacteremia	2
Oliguria	2
Re-admission	2
Atelectasis	2
Hypertensive urgency	1
Partial intestinal obstruction	1
Urinary retention	1
Fluid overload	1
Phlebitis	1
Hypotension	1
Pelvic lymphocele	1
SIRS	1
Second abdominal surgery	1
Lymphedema	1

SIRS, Systemic inflammatory response syndrome.

The patient, who had had SBO due to Meckel's diverticulum, had a second abdominal surgery. On the ninth postoperative day, a tear in the aponeurosis was discovered, resulting in contained evisceration. During surgery, a severe case of adherence syndrome was observed between a small section of the intestines and the subcutaneous tissue, as well as the border of the aponeurosis. However, the anastomosis was found to be in a perfect state.

Two patients presented with late complications, and in both cases, these were attributable to the diverticulum's resection. Between the two patients, one had undergone a single abdominal surgery due to Meckel's diverticulum and presented with partial intestinal obstruction 3 years later, which resolved without the need for surgical intervention. The other patient experienced an episode of ileitis 2 years after surgery and had an episode of high-grade partial intestinal obstruction at the site of anastomosis 25 months after surgery.

In the symptomatic group, eight patients (36%) had already undergone abdominal surgeries prior to experiencing a complication related to Meckel's diverticulum. These surgeries included two appendectomies, two left, one right, and one bilateral inguinal herniorrhaphy, three cholecystectomies, one hysterectomy, and one hepatic transplant. Arguably, if the surgeon had inspected the last meter of the terminal ileum, it is possible that he would have discovered the diverticula, and if resected, it is likely that the complications experienced by these patients could have been prevented. Among the eight patients who underwent diverticulectomy, only one patient experienced medical complications that cannot be attributable to the diverticulum's resection. The patient’s examination revealed an incidental discovery of a mass in the terminal ileum, specifically identified as a neuroendocrine small-cell tumor. As a result, a right hemicolectomy was performed during the same procedure. One patient, who had previously undergone an appendectomy, experienced SBO caused by Meckel's diverticulum, and pathologists stated that the obstruction was due to adhesions that formed around the diverticulum secondary to the previous surgery, rather than being caused by the diverticulum itself.

## Discussion

After reviewing the 27 adult patients with Meckel's diverticulum from the authors’ clinical experience, a total of 22 patients were symptomatic, and SBO was found to be the most common complication. This supports the findings of other authors, who have observed that intestinal obstruction, mainly due to adhesions that involve the diverticula, is the most prevalent clinical complication of Meckel's diverticulum in adults, while hemorrhage is the most common complication in the pediatric population (10). Another finding that correlates positively with other series is the prevalence of males within the symptomatic groups, as Stone et al., Groebli et al., and Leijonmarck et al. (11–13) reported sex ratios of 2:1, 3:1, and 1.8:1, respectively.

Meckel's diverticulum presents with a variety of complications, none of which are clearly matched to the etiology (1, 14). A high level of suspicion is required to direct the examinations toward its diagnosis. The ^99^mTc-pertechnetate scintigraphy, also known as ectopic gastric mucosa scan or “Meckel's scan,” is the primary specific diagnostic method (15). When used correctly, this scan could reach a sensitivity of 86% and specificity of 98% according to some series; nevertheless, several studies conducted in the pediatric population have reported a sensitivity and specificity of 100% (16, 17). Pharmacological sensitization using pentagastrin, ranitidine, cimetidine, glucagon nasogastric aspiration, saline lavage of the bladder, and repeated scan can improve these low rates (17). However, scintigraphy has a high incidence of false negatives because it needs at least 1.8 cm (18) of the gastric mucosa to detect ectopic tissue in the diverticulum, which is, in many cases, absent (18).

In addition, the scintigraphic activity may be reduced by dilution due to bowel hypersecretion or sudden hemorrhage, making it impossible to detect gastric mucosa (18). Two patients, aged 18 and 20 years, were diagnosed preoperatively using this method in this study. Both patients presented with rectal bleeding, and in both patients, ^99^mTc-pertechnetate scintigraphy was performed with a positive result of ectopic gastric mucosa within the diverticulum, making a 100% accuracy and specificity in this study. This was possible due to the surgeon’s high index of suspicion. However, the use of diagnostic modalities depends on the clinical presentation of each patient. When active bleeding is detected, a red blood cell tomography scan is the preferred diagnostic method due to its high sensitivity and specificity, which are both approximately 90% (16, 17).

However, Meckel's diverticulum is often and understandably misdiagnosed, confusing its complications with other pathologies, particularly appendicitis, since it is difficult to differentiate them (19, 20). In this series, 11.7% of the symptomatic patients were diagnosed with appendicitis simultaneously, while 5.8% of this same group were misdiagnosed with Meckel's diverticulum, and 40% of the incidentally found group were found to have appendicitis. The inflammation of Meckel's diverticulum can be more dangerous than appendicitis (20) due to the position of Meckel's diverticulum on the antimesenteric border of the ileum. Simultaneously, the cecum walls and other structures in the right iliac fossa may contain and limit the inflammatory process (21).

Concerning suspected SBO, initial assessment of a patient typically involves plain abdominal films due to their low cost and wide availability (22). According to the Bologna guideline, it is recommended that all patients evaluated for SBO should have one, while CT scans should not be required unless physical examination and plain films are not conclusive (23). However, CT scans help in diagnosing a complete obstruction, elucidate the etiology of SBO, exclude non-adhesional pathology, and determine cases of strangulation (23), while plain x-ray efficiency on these subjects is very limited, if not, futile. An important advantage of CT scans over plain abdominal films is the “increased confidence of identification of the transition zone” that helps determine the etiology of the obstruction (22). The CT criteria for the diagnosis of SBO are divided into major (dilation of small bowel ≥2.5 cm and colon not dilated <6.0 cm; and transition point from dilated to non-dilated small bowel) and minor (air–fluid levels and colon decompressed) (22). In a case series comparing the efficiency between plain abdominal films, ultrasound (US), and abdominal CT scans, Suri et al. (24) concluded that CT scans had much higher sensitivity compared with plain films and the US (93%, 77%, and 83%, respectively). The ease of finding the level of obstruction was also evaluated, with an efficacy rate of 93%, 60%, and 70%, respectively (24). The most striking finding was related to determining the cause of obstruction. The detection rate was 87% among patients who underwent CT scans, compared with 7% in plain films and 23% in the US (24). Two patients in the study did not have SBO, mistakenly diagnosed as having it by the plain film while none with CT scans.

In this series, 54% of symptomatic patients presented with SBO due to Meckel's diverticulum, and in all of the cases, an abdominal CT scan diagnosed the obstruction. However, the diagnosis of Meckel's diverticulum by a CT scan is poses significant challenges (21). The CT pattern of a Meckel's diverticulum is a blind-ended outpouching digestive structure arising from the antimesenteric side of the terminal ileum; this structure is found in the right lower quadrant or lower abdomen and is connected to the small bowel by a neck of varying caliber (25). Previous studies have suggested that diverticular dimensions and symptoms are related. This study found that in the symptomatic patients, the diverticula tended to be longer and with a wider base than that in the incidentally found group. In total, 88.2% (17 patients) of the symptomatic group and 60% of the asymptomatic (three patients) group had a diverticular length greater than 2 cm. Despite the disproportion of the number of patients in both groups and this study includes a small sample size, this finding is similar to the study by Park et al. (26), which showed that patients with diverticula longer than 2 cm had a 2.2-fold higher likelihood of being symptomatic. Despite greater values of length and width in the symptomatic patients, it cannot be stated that these measurements are associated with diverticular complications since it could be argued that the diverticulum may be enlarged by obstruction or inflammation.

It has been reported in several articles that the presence of gastric ectopic tissue in Meckel's diverticulum is associated with symptoms. In this series, 53% of the symptomatic patients had ectopic gastric mucosa within the diverticula, while none of the asymptomatic patients did. After a closer observation, it was found that 100% of the patients who experienced a hemorrhage had fundus and corpus gastric mucosa, one out of two patients with perforation also had it, and 44% of patients with SBO had it as well. The patient with perforation and gastric mucosa also had an ulcer. These results support Park's findings that 43% of the symptomatic patients and 14% of the asymptomatic patients had ectopic mucosa (26, 27).

Surgical management is considered mandatory for symptomatic cases of Meckel's diverticulum, although the appropriate management of incidentally discovered Meckel's diverticula is still controversial. The type of surgical procedure and approach in adult patients also remains to be a matter of debate (9, 28). The surgical option for treating this condition includes diverticulectomy and wedge and segmental resection, with segmental resection considered the most appropriate procedure with high success rates due to a complete resection of the ectopic tissue (9, 28). Some authors categorize the procedure depending on the length of the diverticula and the type of complication. For long diverticula with simple diverticulitis, active bleeding, or an incidental finding, diverticulectomy is the recommended treatment. However, if the diverticula is short (with a height to diameter ratio of <2 cm), wedge and segmental resection is the most effective approach (9, 28). In these cases, it is crucial to avoid incomplete resection of ectopic tissue, which can be determined either by palpation or by diverticula size (9, 28). Some authors such as Ezekian et al. (9, 28) suggest that conventional palpation could be avoided, and minimally invasive approaches can be performed without increasing morbidity and mortality rates, a finding consistent with the study reported by Jung et al. (9, 28), with acceptable complication rates and high success rates, when preoperative ileus and older age are considered as predictors of complications (9, 28). In our study, patients with intestinal obstruction were most frequently treated by laparotomy approach when compared with bleeding, simple diverticula, or incidental findings. This approach resulted in a shorter length of stay, but with no differences in morbidity rate. These findings align with global reports (9, 28).

The limitations of the present study encompass all those associated with descriptive studies. Since cases were not chosen from representative population samples, they cannot generate information on rates, ratios, incidences, or prevalence. Nevertheless, to the best of our knowledge, this is the first study conducted on the Latin American population. It contributes to the existing body of evidence regarding this rare pathology among this specific population.

## Conclusion

Meckel's diverticulum remains to be an uncommon pathology in the adult population. The results of our study show a variety of clinical presentations including bleeding, simple diverticulitis, small bowel obstruction, or incidental finding. The surgical management in our population demonstrates acceptable rates of morbidity and mortality. Nevertheless, the definitive surgical management is still a matter of debate for asymptomatic patients. Further studies are required to confirm our findings.

## Data Availability

The raw data supporting the conclusions of this article will be made available by the authors, without undue reservation.
